# Clival Metastases: Single-Center Retrospective Case Series and Literature Review

**DOI:** 10.3390/jcm13092580

**Published:** 2024-04-27

**Authors:** Alessandro Carretta, Giacomo Sollini, Federica Guaraldi, Arianna Rustici, Marcello Magnani, Sofia Asioli, Marco Faustini-Fustini, Ernesto Pasquini, Matteo Zoli, Diego Mazzatenta

**Affiliations:** 1 Department of Bio-Medical and Neuromotor Sciences (DIBINEM), University of Bologna, 40138 Bologna, Italy; alessandro.carretta2@unibo.it (A.C.); sofia.asioli3@unibo.it (S.A.); matteo.zoli4@unibo.it (M.Z.); diego.mazzatenta@unibo.it (D.M.); 2 Programma Neurochirurgia Ipofisi—Pituitary Unit, IRCCS Istituto delle Scienze Neurologiche di Bologna, 40139 Bologna, Italy; federica.guaraldi@ausl.bologna.it (F.G.); marco.faustini@isnb.it (M.F.-F.); 3 ENT Unit, Bellaria Hospital, Azienda USL Bologna, 40139 Bologna, Italy; giacomo.sollini@ausl.bologna.it (G.S.); ernesto.pasquini@ausl.bologna.it (E.P.); 4 Neuroradiology Unit, IRCCS Istituto delle Scienze Neurologiche di Bologna, Ospedale Maggiore, 40139 Bologna, Italy; arianna.r87@gmail.com; 5 IRCCS Istituto delle Scienze Neurologiche di Bologna, 40139 Bologna, Italy

**Keywords:** clivus, endoscopic endonasal, outcome, complication, metastasis, GEP-NET

## Abstract

**Background/Objectives**: Clivus metastases from distant neoplasms are uncommon occurrences both in clinical practice and the neurosurgical literature. Surgical management is debated, particularly about the role of surgery and the preferable approach. The aim of this study was to report our surgical experience and review the concerning literature. **Methods**: Our institutional registry was retrospectively reviewed, and patients who underwent surgical treatment for clival metastasis from 1998 to 2023 were included. A PRISMA systematic review of the literature was performed. **Results**: Four patients were enrolled, and all of them underwent an endoscopic endonasal approach (EEA). Three presented with cranial nerve (CN) VI palsy. The aim of surgery was biopsy in all cases. No complications were reported. Mean overall survival (OS) was 6 ± 1 months. The systematic review retrieved 27 papers reporting 39 patients who underwent the surgical treatment of clivus metastases. Most of them (79.5%) presented with CN palsies, and EEA was the preferred approach in 92.3% of the cases, to perform a biopsy in most patients (59%). Two hemorrhagic complications (5.1%) were reported, and the mean OS was 9.4 ± 5.6 months. **Conclusions**: Clival metastases are uncommonly observed, in most cases, during advanced stages of oncological disease. The aim of surgery should be the confirmation of diagnosis and symptomatic relief, balancing the risk–benefit ratio in a multidisciplinary context. EEA is the approach of choice, and it should be carried out in experienced tertiary skull base centers.

## 1. Introduction

Clival metastases from distant neoplasms are uncommon occurrences, accounting for 0.02% of intracranial tumors, and they rarely require surgical treatment [1,2]. The available literature concerning these lesions is scarce, with an approximate amount of one-hundred published cases, mostly in scattered series and reports [1,3,4,5].

The location of these lesions can cause a unique range of symptomatology, mainly strictly connected to the anatomical relationship of their arising side (the clival bone), with the cranial nerves (CN) III, IV, V, VI, IX, X, XI, and XII, the overlying cavernous sinus, and the pituitary gland. Conversely, they can be asymptomatic incidental findings detected in the course of neuroradiological examinations for the staging of the primitive neoplasm, as a brain computed tomography (CT) scan, magnetic resonance imaging (MRI), or positron emission tomography (PET). Since there is a lack of specific studies on these lesions, there is no clear consensus about the preferable strategy for their treatment; particularly, no reports have investigated the role of surgery and its impact on patients’ overall survival (OS). Moreover, it has not been considered when surgery is indicated and whether its goal should be limited to a bioptic sampling for histological characterization or should be extended with a resective aim [5].

The aim of this study is to retrospectively analyze our single-center case series of surgically treated clival metastasis in order to consider when surgery can be a valid treatment option and to report its impact on OS. A systematic review of the literature was also performed to discuss indications and nuances of the surgical option for these uncommon neoplasms.

## 2. Materials and Methods

### 2.1. Case Series

Our Institutional database (Programma Neurochirurgia dell’Ipofisi—Pituitary Unit, IRCCS Istituto delle Scienze Neurologiche, Bologna, Italy), ranging from 1998 to December 2023, was retrospectively reviewed to include all the consecutive cases of surgically treated clivus metastases. Inclusion criteria consisted of a histological confirmation of the diagnosis of metastasis and availability of preoperative medical reports and complete follow-up. Patients who had not undergone surgery or were lost at follow-up were excluded. 

All the patients underwent a preoperative complete neurological examination endocrinological basal assessment of pituitary function and, when necessary, a brain contrast-enhanced MRI and CT with angiography sequences and full-body imaging (CT or PET) according to their oncological history. Every case was discussed by a dedicated multidisciplinary board, composed of neurosurgeons, ENT surgeons, pathologists, radiologists, oncologists, radiotherapists, and radiosurgeons, to confirm the surgical indication and its aim (bioptic or resective). The surgical procedure was performed via an endoscopic endonasal approach in all cases, with the intraoperative implementation of electromagnetic neuronavigation. Clinical course and complication were retrieved from the electronic records, and complications were defined as any deviation from the normal postoperative course [6]. All the patients underwent a head CT scan at 6 h after the procedure to exclude early complications and an MRI within 72 h to assess the extent of resection (EOR). Gross total resection (GTR) was defined by a senior neuroradiologist as the complete resection of pathologic tissue at postoperative imaging, while subtotal resection (STR) was defined as the presence of any neoplastic remnant. Further adjuvant therapies (chemotherapy and radiotherapy) were indicated according to multidisciplinary evaluation and neoplasm histotype. Further brain MRI imaging was repeated every three months after the procedure, alongside clinical assessments. The overall survival (OS) of every included patient was also gathered.

The patients signed their informed consent for the scientific anonymized use of intraoperative and radiological images.

### 2.2. Literature Review

#### 2.2.1. Search Strategy 

A systematic literature review of the last 25 years was performed in accordance with the Preferred Reporting Items for Systematic Reviews and Meta-Analyses (PRISMA) statement guidelines [7]. MEDLINE and SCOPUS databases were queried using individual keywords. Two purposely defined search strings were used for MEDLINE search: (“clivus” AND “metastasis”) and (“clival” AND “metastasis”). The results were then limited to the English language and human subjects. After duplicate removal, the titles and abstracts were first screened and, for the papers deemed appropriate, full texts were obtained and reviewed for appropriateness and the extraction of data. The articles’ reference lists were examined to identify any other relevant studies. The individual steps of title and abstract screening, full-text review, and data extraction were performed independently by two reviewers (A.C. and M.M.); disagreements at any stage were resolved by discussion and consensus, and the senior authors reviewed and approved the selection. The systematic review was not registered in any online database. The last search was performed on 29 February 2024.

#### 2.2.2. Selection Criteria

The inclusion criterion was the report of any metastatic lesion of a distant neoplasm involving the clivus which underwent any type of surgical procedure, with any approach, either with a resective or a bioptic aim. The exclusion criteria were as follows: clivus involvement by contiguous locoregional advanced tumors (i.e., nasopharyngeal carcinoma or other analogous ENT neoplasms), extensive skull base neoplastic disruption with only secondary clivus involvement, and lack of data concerning type of surgical approach and complications. 

#### 2.2.3. Data Extraction

Data from the included studies were extracted, organized, and analyzed using Microsoft Excel 2019 (Microsoft Corp, Redmond, WA, USA). The collected variables included the first author, publication year, tumor location, tumor histopathology, patients’ symptoms, type of approach, extent of resection and complications, symptoms outcome, tumor recurrence/progression at follow-up, complementary therapies, and mortality.

## 3. Results

### 3.1. Case Series

Our series is composed of four cases: three were males, and the average age was 69.5 ± 15.3 years. The diagnosis of clival metastasis was preoperatively suspected in two cases, as the primitive tumor was already diagnosed, while in the other two, this was detected only after the skull base surgery. No patients were lost at follow-up. As reported in Table 1, the primitive tumor was a lung carcinoma in two cases (50%), and a gastric signet ring cell carcinoma and gastroenteropancreatic neuroendocrine tumor (GEP-NET) in the other four cases. All of them underwent operations through an endoscopic endonasal approach.

In three cases, the clinical presentation was a CN VI palsy, while in the remaining case, the detection of clival metastasis was an incidental finding in the course of a PET for GEP-NET staging. The patients’ average preoperative Karnofksy performance status (KPS) was 80 ± 8.2. The average lesions’ volume was 4.3 ± 2.6 cm^3^. Tumors were located in the middle third of the clivus in three cases, with the remaining one located in the superior two-thirds, and all were completely extradural.

The aim of surgery was bioptic in three patients. In one case (patient #3, Table 1), the preoperative neuroradiological suspect was of a clivus chordoma; therefore, the procedure was planned with a resective aim. However, as the intraoperative histological analysis was suggestive of metastasis, any further resective maneuvers were halted. The subsequent body CT demonstrated lung carcinoma. The mean hospital stay was 2 ± 1 days. Patients started oral feeding the same day of surgery and were mobilized after 12 h. No surgical complications or 30-day mortality were observed in the series.

At follow-up, patients’ preoperative CN VI palsy was stable in three cases, while the neurologically intact patient developed no further symptoms. Three patients underwent further systemic chemotherapy according to specific protocols targeting the primary neoplasm and locoregional conventional radiotherapy (RT). Patient #4 subsequently underwent resection of further breast metastasis (as extensively reported in the illustrative case) and then was treated with locoregional RT and systemic chemotherapy. At her last follow-up, no locoregional progression was observed. Three patients died after an average timespan of 6 ± 1 months due to systemic progression of the disease, while the last patient was still alive at 3 months follow-up.

### 3.2. Illustrative Case

A healthy 47-year-old woman (patient #4, Table 1) with an otherwise unremarkable clinical history presented in the emergency department complaining of recurrent vomit episodes. Her abdominal CT revealed a contrast-enhancing circumferential thickening of a distal ileum loop (with an extension of 5 cm), with concurrent sub-occlusive dilation of the cranial loops and mesenteric lymphadenopathy (maximal diameter 2.5 cm). Further MR enterography confirmed the pathological findings, inferring the clinical suspect of a GEP-NET. Therefore, the patient underwent Gallium-68 DOTANOC positron emission tomography (PET), which showed pathologic somatostatin receptor uptake at different levels: small bowel and mesenteric lymph nodes as expected, skull base (SUVmax: 30.9), and left breast (SUVmax: 4.1). Blood tests and the 24 h urine test with serotonin and 5-HIAA dosing were unremarkable. The patient then underwent a laparotomy with distal Ileum resection, local lymphoadenectomy, and latero-lateral ileum anastomosis. The histopathological analysis revealed a G2 neuroendocrine tumor (NET) with complete infiltration of the intestinal wall and adjacent loop invasion, with lymphovascular and perineural invasion and a Ki-67 index of 4.1%. The TNM/AJCC eighth edition stage was pT4, pN1 [8]. The left breast pathologic uptake was caused by an 8 mm large hypoechoic lesion at ultrasonography. Needle biopsy showed a tissue compatible with ductal infiltrative carcinoma, despite detailed analysis being precluded by the scarcity of the specimen. 

**Figure 1:**
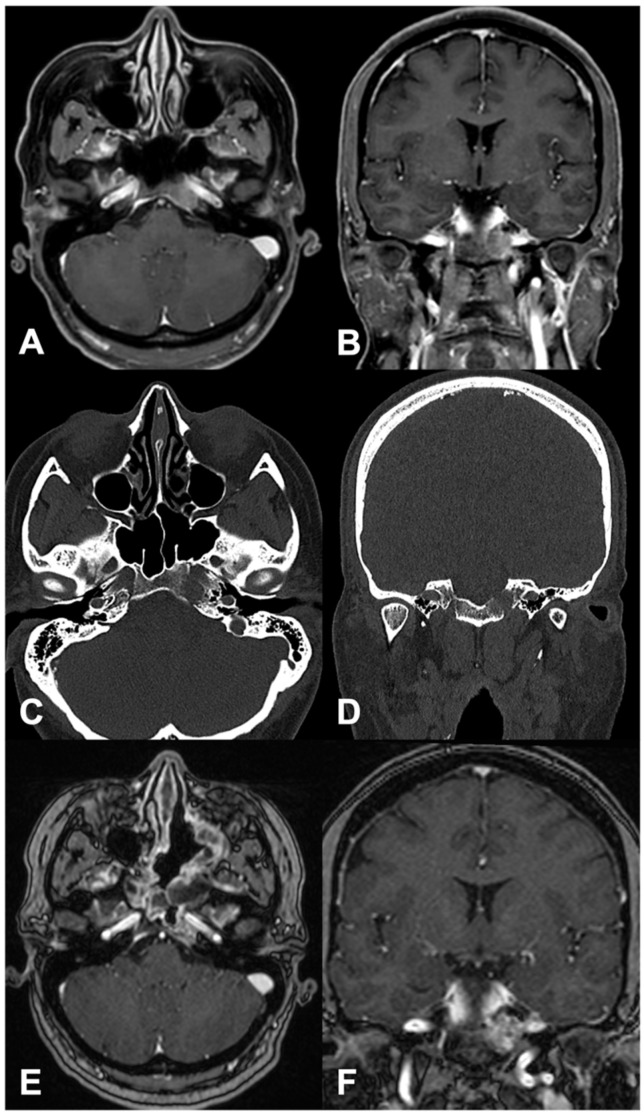
Preoperative images of case #4 (axial and coronal T1-weighted contrast-enhanced MRI, (**A**,**B**); axial and coronal bone window thin-slice CT, (**C**,**D**)). The neuroimaging shows a contrast-enhancing lesion on the left side of the clivus, lying posteriorly to the left intrapetrous ICA (**A**). The CT reveals the lesion to be osteolytic, with cortical bone interruption anteriorly and posteriorly, suggesting its invasiveness of the posterior cranial fossa and carotid canal (**C**). The 3-month postoperative images (axial and coronal T1-weighted contrast-enhanced MRI, (**E**,**F**)) show the partial resection of the medial part of the neoplasm.

Contrast-enhanced brain MRI and CT were performed, revealing a 10 × 11 × 12 mm contrast-enhancing osteolytic left paramedian clival lesion (Figure 1).

Furthermore, the tumor, located dorsally to the C2 and C3 segments of the left internal carotid artery (ICA), showed anterior and posterior cortical bone thinning interruption, consistent with possible posterior cranial fossa and carotid canal involvement. The localization was suspected to be consistent with clival metastasis, but surgical biopsy was suggested. The case was discussed by our institutional multidisciplinary oncological board, and a biopsy of the skull base lesion was advised. 

**Figure 2:**
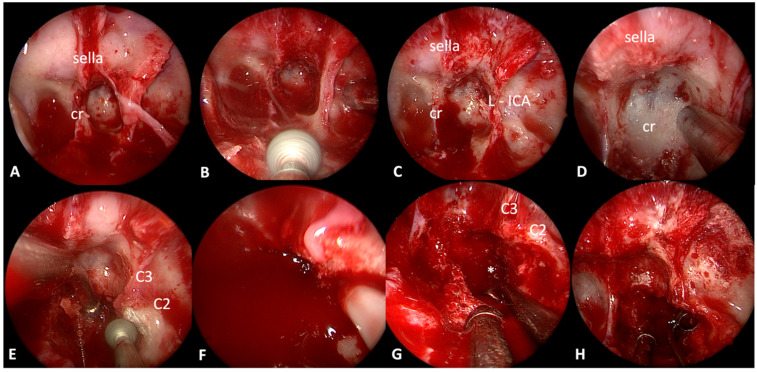
Intraoperative endoscopic endonasal images. A standard binostril endoscopic endonasal approach is performed, with posterior septostomy and anterior sphenoidotomy (**A**). The floor of the sphenoidal sinus is then drilled, as well as the intrasinusal septa (**B**), to expose the anterior aspect of the clivus and the paraclival arteries (**C**). Tumor location and left ICA position were checked with intraoperative navigation (**D**,**E**) before starting to drill the ventral aspect of the clival bone covering the tumor. An intraoperative Doppler probe was used to localize the ICA course (**F**). The tumor (*), which macroscopically appears as a fleshy reddish mass in the solid clival bones, is then partially resected with curettes and suction (**G**,**H**). C2: petrous internal carotid segment, C3: lacerum internal carotid artery, cr: clival recess, L-ICA: left internal carotid artery.

The surgical procedure was performed in semisitting position, through a binostril endoscopic endonasal approach (EEA) using a 2D HD camera (Spies™, Karl Storz SE, Tuttlingen, Germany). After the implementation of AxiEM™ magnetic neuronavigation merging MR and CT images (StealthStation™ S8, Medtronic, Minneapolis, MN, USA), posterior septostomy and anterior sphenotomy were performed. The floor of the sphenoid sinus was removed with cutting rongeurs and a high-speed drill to completely expose the clival recess and the paraclival ICAs bony prominences (Figure 2).

With the aid of neuronavigation, the osteo-erosive lesion of the clivus was identified. The tumor, appearing as a solid blood-oozing reddish mass, was debulked with angled aspirators and curettes, and samples were gathered. During these maneuvers, utmost care was used to prevent any possible injury to the left ICA, especially its C2–C3 genu lying directly anteriorly, systematically checking its position with a Doppler probe (Med-Europe SRL, Bologna, Italy). The dura mater of the posterior cranial fossa was exposed during the debulking steps. The resection was interrupted in the most lateral aspect of the mass, posteriorly to C2–C3 genu, to avoid any possible unnecessary vascular injury. Hemostasis was then achieved with bipolar coagulation, gel foam, and thrombin matrix. The bony defect was covered with a middle turbinate mucoperiosteum graft (Figure 2). The further clinical course was unremarkable, and the patient was discharged at home on the second postoperative day. Histopathological analysis revealed atypical epithelial cells with nidal disposition and bone infiltration, a Ki-67 index of 4%, and showed immunohistochemistry positivity for Cam 5.2, Chromogranin A, CDX-2, and the somatostatin 2A receptor. These findings were consistent with the GEP-NET metastasis.

The patient then underwent a quadrantectomy two months later to resect the breast tumor, which resulted in a further secondary localization of the primary GEP-NET. At 3 months follow-up, the patient was alive with an unremarkable clinical status. MRI showed no locoregional progression in the clival region (Figure 1), and a Gallium-68 DOTANOC PET did not reveal any new-onset localizations. Adjuvant therapies were therefore started with skull base locoregional RT and systemic chemotherapy. 

Of note, to the best of our knowledge and according to the included systematic review of the literature, this is the first report of a clival metastasis of a GEP-NET.

### 3.3. Literature Review

**Figure 3:**
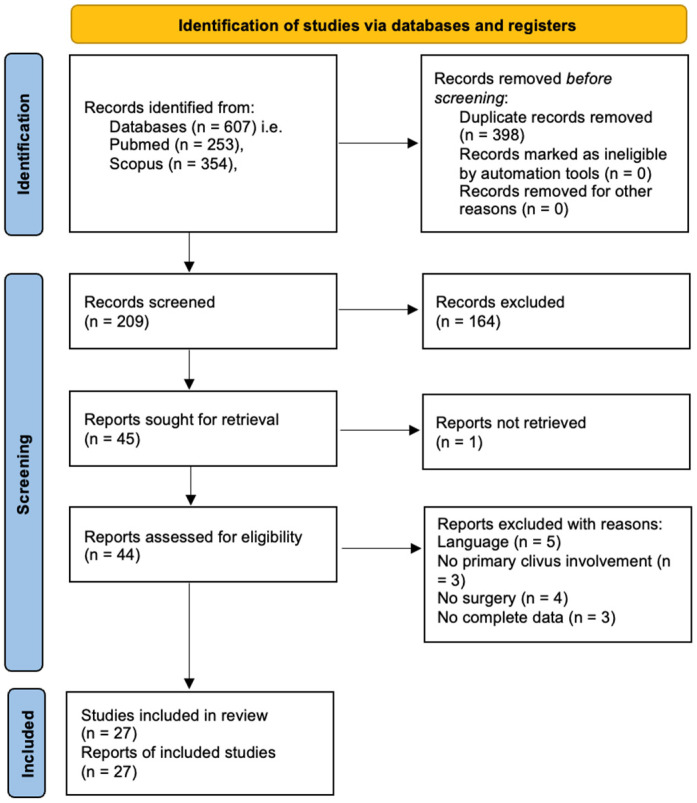
PRISMA flowchart of the included systematic literature review.

The literature review retrieved 27 studies, reporting 39 cases of surgically treated clivus metastases (excluding the reported cases), outlined in Table 2 and Figure 3.

Of those cases, 22 were males (56.4%) and 17 were females (43.6%). Average age was 58.1 ± 17.2 years. Primary tumor histology was heterogeneous, with prostate cancer as the most common occurrence (8 cases, 20.5%), followed by breast (6, 15.4%) and lung carcinoma (4, 10.3%). Rarer findings were hepatocellular carcinoma (3, 7.7%), renal clear cell (2, 5.1%), and thyroid carcinoma (2, 5.1%). All the other histotypes reported in Table 2 were anecdotal. 

All the reported patients were symptomatic, in most cases (31 patients, 79.5%), with ophthalmoplegia due to third, fourth, and/or sixth cranial nerve deficits or other CN palsies. Surgery was performed in most of the cases with an EEA (36: 92.3%), even if anecdotal mentions of petrosectomies or cervical approaches were reported. The goal of surgery was a biopsy in most cases (23, 59%), followed by partial or subtotal resections in 11 patients (28.2%). GTR was reported only in five cases (12.8%). Among the 16 patients who underwent resective procedures, clinical outcome regarding symptomatic relief was reported in seven cases. Five of these cases (71.4%) experienced some degree of symptomatic improvement after the procedure. Complications were rare, with only two (5.1%) cases of hemorrhage reported. 

**Figure 4:**
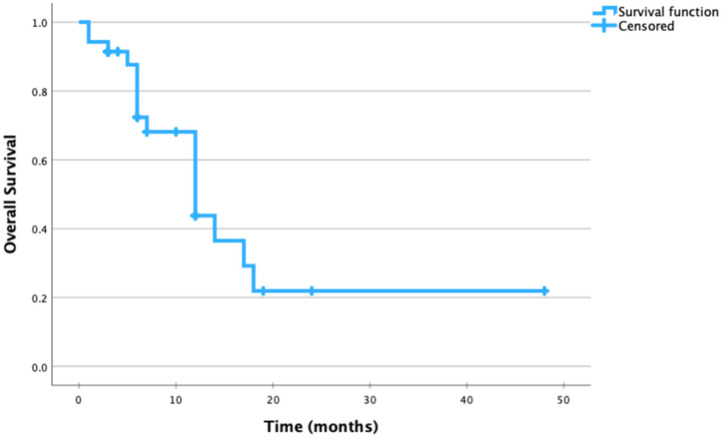
Kaplan–Meier overall survival plot of all the reported patients with available data.

Follow-up and survival data were reported in 31 cases; of these, 17 patients were alive after an average follow-up of 10.5 ± 11.5 months. The other 14 patients showed an average OS of 9.4 ± 5.6 months (Figure 4).

## 4. Discussion

Our study shows that clival metastases rarely require surgery, with only 39 cases reported in the literature, excluding the four additional patients of this report. Indeed, the occurrence of clival metastasis is likewise uncommon, with 58 cases reported in a recent systematic review of the literature with a timespan comparable to our study, occurring especially in advance-staged disease and, therefore, rarely requiring surgery [1]. It was our aim to expand and amend the available literature reports, adding our clinical case series and focusing exclusively and in detail on surgical treatment, as never previously performed [1]. In our series, patients had a mean age of 69.5 ± 15.3, with limited comorbidities and high KPS (mean 80 ± 8.2) at diagnosis, similarly to the cases reported in the literature, presenting a mean age of 58.1 ± 17.2. The main aim of surgery is the confirmation of the histopathological suspect [5], from the possible differential diagnosis of the tumor of the region, ranging from chordomas and chondrosarcomas to plasmacytomas or other hematological disorders, with the goal of guiding further adjuvant therapies, as occurred in patient #4 of our series. As expected, the most common primary tumor histology was prostate cancer (20.5%). The propensity of prostate cancer to spread to distant bones is well known in clinical practice and investigated in the medical literature, given the peculiar interactions between cancer cells and bone microenvironment, and the clivus does not seem to be excluded [33,34]. Our literature review also confirms this hypothesis since biopsy was the aim of surgery in the majority of cases (59%). A further surgical indication is the debulking of the neoplasm to alleviate the symptomatology. Indeed, in the literature, 79.5% of the operated patients had some degree of CN palsy. In these cases, resective debulking surgery could effectively decompress the neural structures, and the literature reports five patients (accounting for 71.4% of the cases where these data were reported, albeit this result could be overestimated, and improvements were in some cases marginal) who experienced any degree of improvement in these preoperative neurological deficits. However, it should be remarked that whether the deficit is due to nerve infiltration by the tumor, chances of postoperative recovery are poor. Moreover, since the possible infiltration is also of vascular structures, such as ICA, clival metastasis full resection can be harmful for the risk of intraoperative or delayed bleeding or of permanent neural deficits. 

EEA has been revealed to be an excellent approach for clival metastasis. This approach mainly relies on the well-known established experience achieved in the treatment of clival chordomas [35,36,37,38,39,40]. Indeed, in the literature, EEA resulted in being the preferred approach for the treatment of clival metastases, chosen in more than 90% of cases. Most of these lesions are extradural, and this approach allows the surgeon to ventrally reach the tumor, with no need for demolitive or aggressive approaches, brain retraction, or neurovascular structures manipulation. As a consequence, tumor biopsy and eventually debulking can be effectively achieved in all cases, with very limited morbidity, consisting only of one case of hemorrhage as the only reported complication of 36 surgical procedures, while in our series, no complications were described. Thanks to its minimally invasiveness, this approach is characterized by a fast patient recovery, with immediate restoration of spontaneous breathing, oral feeding, and mobilization. Therefore, it favors the following adjuvant therapies, since it permits to effectively preserve the patients’ quality of life, with a reduced risk of major complications, possibly hampering their postprocedural recovery. Concurrently, the flexibility of EEAs allows the surgeon to preoperatively plan, or even intraoperatively adapt, the surgical strategy to each patient, while other described approaches, such as anterior petrosal and cervical transjugular, are less flexible and more demolitive, exposing the patient to a higher risk of complications [11,32].

In one case (patient #3), we observed a clival metastasis radiologically mimicking a chordoma, thus, combined with the patient’s negative oncological history, misguiding the surgical strategy, which was planned with a demolitive aim. The intraoperative findings then steered the surgical strategy to a more conservative aim. In those cases characterized by uncertain differential diagnosis, it is in our opinion that the mandatory routinary use of intraoperative histopathological examination could provide relevant information to guide the further surgical steps.

Further, the OS results of our series were similar to those reported in the literature, which, in our cases, dramatically shortened to an average of 6 months when cranial nerve involvement was manifest [41]. Therefore, the involvement of cranial nerves (reported in our review in almost 80% of cases) is per se a detrimental prognostic factor, as previously stated [41] and confirmed by our experience, as all of the reported patients showing cranial nerve (75% of our case series) involvement passed away after an average of 6 months despite systemic aggressive multimodal therapy. Consequently, we would like to remark that surgical indications to a clival metastasis should be strict, selecting only those patients with a potential clinical or oncological positive impact from surgery, as symptoms alleviation or indications for further adjuvant treatments, especially avoiding potential overtreatment in advanced or already functionally compromised patients. Thus, the indication for treatment should be based on a careful risk–benefit balance. An alternative therapeutic option can be locoregional RT, which could be reserved for more fragile patients, not suitable for surgery, with a possible symptomatic relief, as demonstrated by Sturgis et al. [1,3]. Radiosurgery could also be considered as an alternative or complementary treatment option, proving to be safe and effective, as recently reported by Huq et al. [42]. We claim, therefore, that the management strategy of cases of clival metastases should be carefully evaluated and discussed in a dedicated skull base multidisciplinary board, considering all the available treatments and every single feature, from the patient’s clinical status to the neoplasms’ extension and their molecular profile. 

### Strengths and Limitations

The main strength of this paper is that it is the first to purely focus on the role of surgery, and of EEA in particular, for clival metastases. It is also the first description in the literature of a clivus metastasis of a GEP-NET. Moreover, we outlined our surgical strategy and experience in the treatment of those patients based on the experience developed in a tertiary referral center for skull base diseases. Despite the observational retrospective design of our research, no patients were excluded for lacking data or were lost at follow-up.

Nonetheless, this study shows unavoidable limitations. First of all, clival metastases are uncommon, precluding us or other researchers to describe larger case series (possibly prospective or multicentric), on which a more accurate analysis of the complication rate, outcome predictive features, and management strategies could be based. Moreover, literature reports are similarly scarce, with a significant heterogeneity of the primary neoplasms, which could present clival metastasis during their clinical course, and of the histotype-tailored management of adjuvant strategies (especially chemotherapy and, possibly, immunotherapy), which makes a proper OS analysis impossible. The severity of the disease, not uncommonly characterized by a malignant and a quickly aggressive clinical course, has also often precluded authors from reporting PFS, making its analysis also impossible.

## 5. Conclusions

Clival metastases are relatively uncommon tumors, observed especially in the advanced stage of oncological diseases. Surgery is rarely needed, mostly to confirm the preoperative clinical suspect or in the case of non-evident primitive tumors at diagnosis, guiding further adjuvant therapies, or with the aim of alleviating patients’ neurological symptoms. 

EEA should be the first option for the surgical management of clival metastases, as it is very well tolerated by patients, with quick recovery, an acceptable morbidity, and rare complications, thus preserving overall quality of life. 

The management of these patients should be carried out in specialized centers and evaluated by multidisciplinary boards, carefully balancing the risk–benefit ratio. Further, well-conducted and designed studies are warranted to identify the optimal multimodal treatment for clival metastases.

## Figures and Tables

**Table 1:** Clinical, radiological, histopathological, and surgical features of the patients reported in our cohort, completed by survival time after the surgical procedure. CN: cranial nerve; CT: chemotherapy; EEA: endoscopic endonasal approach; EOR: extent of resection; GEP-NET: gastroenteropancreatic neuroendocrine tumor; LC: lung carcinoma; PR: partial resection; RT: radiotherapy.

# Patient	Age and Sex	Preoperative Symptoms	KPS	Surgical Objective	Volume (cm^3^)	Clivus Location	Surgical Approach	Skull Base Reconstruction	EOR	Complications	Histology	Adjuvant Therapies	Status at Follow-up	OS (Months)
1	81, M	Right VI CN palsy	70	Biopsy	5.9	Extradural, middle third	EEA	Mucoperiosteum graft	Biopsy	No	LC, previously diagnosed	CT and RT	Deceased	6
2	76, M	Left VI CN palsy	80	Biopsy	4.4	Extradural, middle third	EEA	Mucoperiosteum graft	Biopsy	No	Gastric signet ring cell carcinoma, first diagnosis	CT and RT	Deceased	5
3	74, M	Left VI CN palsy	80	Resection	6.3	Extradural, superior, and middle thirds	EEA	Pedicled flap	Biopsy	No	LC, first diagnosis	CT and RT	Deceased	7
4	47, F	Intact	90	Biopsy	0.6	Extradural, middle third	EEA	Mucoperiosteum graft	PR	No	GEP-NET, previously diagnosed	Resection of breast metastasis	Alive	Alive at 3 months

**Table 2:** Summary of included studies, describing preoperative features, primary neoplasms, type of surgical approach, extent of resection (EOR), and complications. BC: breast carcinoma; CN: cranial nerve; CT: chemotherapy; EEA: endoscopic endonasal approach; EOR: extent of resection; GEP-NET: gastroenteropancreatic neuroendocrine tumor; GI: gastrointestinal; GTR: gross total resection; HCC: hepatocellular carcinoma; LC: lung carcinoma; NA: not available; OS: overall survival; PC: prostate carcinoma; PR: partial resection; RCCC: renal clear cell carcinoma; RT: radiotherapy; STR: subtotal resection; TC: thyroid carcinoma.

Authors	Year	Age and Sex	Primary Neoplasm	Clivus Lesion Volume (cm^3^)	Symptomatology	Surgical Approach	EOR	Complications	OS
Present study	2024								
Patient 1		81, M	LC	5.9	Right CN VI palsy	EEA	Biopsy	No	6
Patient 2		76, M	Gastric signet ring cell carcinoma	4.4	Left CN VI palsy	EEA	Biopsy	No	5
Patient 3		74, M	LC	6.3	Left CN VI palsy	EEA	Biopsy	No	7
Patient 4		47, F	GEP-NET	0.6	Asymptomatic (PET finding)	EEA	PR	No	Alive at 3 months
Ravnik et al. [9]	2020								
Patient 1		67, M	PC	NA	NA	EEA	GTR	No	12
Patient 2		58, F	BC	NA	NA	EEA	GTR	No	12
Douglas et al. [10]	2020								
Patient 1		81, M	PC	17.1	Right CN VI palsy	EEA	Biopsy	No	Alive at 3 months
Cathel et al. [5]	2019								
Patient 1		65, M	HCC	NA	Headache, ophthalmoplegia	EEA	Biopsy	No	NA
Mishima et al. [11]	2019								
Patient 1		76, F	HCC	NA	Right CN VI palsy	Anterior petrosal approach	STR	Hemorrhage	6
Zhang et al. [12]	2018								
Patient 1		54, M	RCCC	NA	Right CN III, IV, V2 and VI palsy	EEA	PR	Hemorrhage	Alive at 4 months
Machìo-Castellò et al. [13]	2017								
Patient 1		59, M	PC	NA	Left CN III, IV, VI palsy	EEA	Biopsy	No	Alive at 12 months
Dekker et al. [4]	2017								
Patient 1		67, M	Duodenal mucinous adenocarcinoma	15.4	Bilateral CN VI palsy	EEA	Biopsy	No	Alive at 24 months
Ho et al. [14]	2017								
Patient 1		60, M	Merkel cell skin carcinoma	NA	Right ophthalmoplegia, facial hypoesthesia	EEA	Biopsy	No	Alive at 6 months
Zagzoog et al. [15]	2017								
Patient 1		43, F	Myxoid liposarcoma	20.2	Right CN VI palsy	EEA	STR	No	Alive at 3 months
Yari et al. [16]	2016								
Patient 1		45, F	TC	12.9	Right CN VI palsy	EEA	STR	No	Alive at 6 months
Tsunoda et al. [17]	2016								
Patient 1		76, F	BC	NA	Dizziness	EEA	Biopsy	No	Alive at 48 months
Kapoor et al. [18]	2015								
Patient 1		35, F	BC	13.2	Left CN VI palsy	EEA	Biopsy	No	Alive at 3 months
Lee et al. [19]	2015								
Patient 1		42, F	Gastric carcinoma	NA	Right CN III, VI palsy	EEA	Biopsy	No	6
Mendelson et al. [20]	2015								
Patient 1		59, F	RCCC	NA	Left CN III, IV, V2 and VI palsy	EEA	STR	No	NA
Zacharia et al. [21]	2015								
Patient 1		60, F	TC	NA	Left CN VI CN palsy, headache, tinnitus	EEA	GTR	No	Alive at 12 months
Patient 2		72, M	LC	NA	Neck pain	EEA	PR	No	1
Patient 3		77, M	PC	NA	Headache	EEA	STR	No	3
Kendre et al. [22]	2014								
Patient 1		34, M	Rectal signet cell carcinoma	NA	Bilateral CN VI palsy	EEA	GTR	No	NA
Vellutini et al. [23]	2014								
Patient 1		54, F	BC	NA	CN XII palsy	EEA	Biopsy	No	NA
Patient 2		64, F	BC	NA	CN VI palsy	EEA	Biopsy	No	NA
Patient 3		79, M	PC	NA	CN VI palsy	EEA	Biopsy	No	NA
Deconde et al. [24]	2013								
Patient 1		62, F	Leiomyosarcoma	3.1	Headache, CN VI palsy	EEA	Biopsy	No	Alive at 7 months
Patient 2		52, F	BC	4.6	Right CN VI palsy	EEA	GTR	No	Alive at 19 months
Bohnstedt et al. [25]	2012								
Patient 1		14, F	Myoepithelial sarcoma	NA	Left CN VI palsy	EEA	STR	No	12
Fukushima et al. [26]	2012								
Patient 1		64, M	Gastric signet ring cell carcinoma	NA	Headache, bilateral CN VI palsy	EEA	PR	No	Alive at 10 months
Baeg et al. [27]	2011								
Patient 1		70, M	GI stromal tumor	NA	Right CN VI palsy	EEA	STR	No	Alive at 3 months
Fraser et al. [28]	2010								
Patient 1		72, M	Carcinoma (unspecified)	NA	Asymptomatic	EEA	Biopsy	No	NA
Kolias et al. [29]	2010								
Patient 1		64, M	PC	NA	Left CN III, IV, V1, V2, VI, VII, IX, X and XII palsy	EEA	Biopsy	No	17
Pallini et al. [2]	2009								
Patient 1		31, M	Melanoma	NA	Left CN VI palsy	EEA	Biopsy	No	18
Patient 2		67, F	LC	NA	Right CN VI palsy	EEA	Biopsy	No	12
Patient 3		69, M	HCC	NA	Left facial pain, right CN III palsy	EEA	Biopsy	No	Alive at 3 months
Patient 4		50, M	PC	NA	CN VI palsy	EEA	Biopsy	No	6
Patient 5		57, M	LC	NA	Headache, CN VI palsy	EEA	Biopsy	No	12
Patient 6		76, M	Squamous cell carcinoma	NA	CN VI palsy	EEA	Biopsy	No	14
Patient 7		70, M	PC	NA	CN VI palsy	EEA	Biopsy	No	Alive at 3 months
McGirt et al. [30]	2005								
Patient 1		3, M	Adrenal neuroblastoma	23.3	Vision loss	Endoscopic ethmoidectomy	Biopsy	No	NA
Ulubas et al. [31]	2005								
Patient 1		51, F	LC	15.8	Headache, left shoulder and neck pain, weight loss	EEA	Biopsy	No	1
Alessi et al. [32]	2003								
Patient 1		66, F	Leiomyoma	NA	Right CN VI and XII palsy	High cervical transjugular approach	STR	No	Alive at 12 months

## Data Availability

The authors declare that the gathered data included and used for the analysis outline are available in the manuscript. Further datasets are available upon reasonable request from the corresponding author.
